# Implementation of nationwide screening of pregnant women for HTLV-1 infection in Japan: analysis of a repeated cross-sectional study

**DOI:** 10.1186/s12889-020-09258-4

**Published:** 2020-07-22

**Authors:** Naohiro Yonemoto, Shunji Suzuki, Akihiko Sekizawa, Shinichi Hoshi, Yoko Sagara, Kazuo Itahashi

**Affiliations:** 1 Department of Neuropsychopharmacology, National Center of Neurology and Psychiatry, 4-1-1, Ogawahigashi, Kodaira, Tokyo, Japan; 2 Department of Public Health, Juntendo University School of Medicine, 2-1-1 Hongo, Bunkyo, Tokyo, Japan; 3 Japan Association of Obstetricians and Gynecologists, 14 Yahatacho, Ichigaya-Hachimanmachi, Shinjuku-ku, Tokyo, 162-0844 Japan; 4 Department of Obstetrics and Gynecology, Japanese Red Cross Katsushika Maternity Hospital, 5-11-12 Tateishi, Katsushika-ku, Tokyo, 124-0012 Japan; 5 Department of Obstetrics and Gynecology, Showa University School of Medicine, 1-5-8 Hatanodai, Shinagawa-ku, Tokyo, 142-8666 Japan; 6 Department of Pediatrics, Showa University School of Medicine, 1-5-8 Hatanodai, Shinagawa-ku, Tokyo, 142-8666 Japan

**Keywords:** Human T-lymphotropic virus type 1, Pregnant, Screening

## Abstract

**Background:**

Screening of pregnant women carrying human T-lymphotropic virus type 1 (HTLV-1) has a crucial role in reducing the number of HTLV-1 carriers. A national HTLV-1 screening program for pregnant women was started in 2011 in Japan. The purpose of this study is to report on the implementation of this nationwide screening program.

**Methods:**

This was a retrospective repeated cross-sectional study. We used datasets from surveys of HTLV-1-antibody-positive pregnant women performed by the Japan Association of Obstetricians and Gynecologists in 2011, 2013, and 2016. Outcomes for evaluation included the number of persons (pregnant women) who conducted the screening test, the number of positive persons (women) identified by these tests, and the proportion of positive persons to the number of persons (women) who conducted the tests.

**Results:**

Numbers of target facilities changed yearly: 1857 in 2011, 2544 in 2013, and 2376 in 2016. The mean number of screening-test participants increased per facility, but the median increased or decreased. The mean number of positive individuals identified decreased. Multivariate analysis results revealed the number of screenings was slightly reduced yearly, although areas (Kanto and Kinki) and high volume in facility types increased. Regarding the positive rates, some areas (Hokkaido/Tohoku, Kanto, and Chugoku/Shikoku) exhibited decreases or increases by facility type. The number of western blotting (WB) implementations decreased in 2016, positive rates identified by WB decreased in 2016 in all areas, and the number of facility types increased. The number of PCR participants increased in 2016 in Kanto and Kinki, but a decrease in facility type was observed. Positive rates were decreased in all areas (except the central region) but facility types were increased.

**Conclusions:**

The nationwide screening program for HTLV-1 in Japan was almost fully implemented. However, regional variations in screening tests were observed during this implementation. Thus, some incentives are needed to encourage proper implementation across all regions.

## Background

Human T-lymphotropic virus type 1 (HTLV-1) infects lymphocytes, a type of white blood cell. HTLV-1 causes adult T-cell leukemia/lymphoma, HTLV-1-associated myelopathy, HTLV-1 uveitis [1], and infective dermatitis [2]. Although these HTLV-1-related diseases can develop in HTLV-1-infected persons, most patients are asymptomatic carriers [1].

HTLV-1 is endemic in areas such as southwestern Japan, the Caribbean, Central and South America, intertropical Africa, and the Middle East [3]. HTLV-1 is sexually, parenterally, and vertically transmissible [4]. Detection of pregnant women carrying HTLV-1 is crucial for reducing the number of HTLV-1 carriers because HTLV-1 is primarily transmitted vertically from mother to child. If this epidemiological trend remains, the implementation of a prenatal screening program will be an important public policy in Japan. This must be reinforced by the authors. Mother-to-child transmission (MTCT) of HTLV-1 occurs mainly via breast milk and refraining from breastfeeding was shown to be effective at reducing MTCT [5–8]. An epidemiological study in Japan reported that breastfeeding was the main route of HTLV-1 transmission [9]. Indeed, the expected outcome of withholding breastfeeding is a reduction of the MTCT rate from 15 to 20% to 2–3% [6]. Because ATL likely develops after a long incubation period of more than 20 years in HTLV-1 carriers via MTCT, the prevention of milk-borne transmission is the most efficient and feasible way to reduce the disease burden.

In Japan, HTLV-1 carriers and individuals with related diseases are particularly prevalent in the southwest region, including Kyushu and Okinawa. However, surveys performed in 2006 and 2007 revealed that carriers have migrated to areas within large cities [10–13]. In response, the Ministry of Health, Labour and Welfare (MHLW), Maternal and Child Health Section passed a notice in November 2010 for an HTLV-1 antibody screening test for pregnant women, which was initiated in 2011.

The purpose of this study was to report on the implementation of the nationwide screening for HTLV-1 in pregnant women conducted since 2011.

## Methods

### Nationwide screening and tests

The Japanese MHLW decided to financially support blood testing for the screening of HTLV-1 in pregnant women in 2010. Specifically, the migration of Japanese people from Kyushu to metropolitan areas was thought to contribute to a significant decrease in HTLV-1 carriers in Kyushu and an increase in Kanto (including Tokyo). Local prefectural governments were responsible for the implementation of the screening. The local governments collaborated with stakeholders and endorsed the screening program. Japanese Clinical Guidelines for Obstetric Practice (edited in 2011 by the Japan Society of Obstetrics and Gynecology and Japan Association of Obstetricians and Gynecologists) recommended carrying out a screening test for anti-HTLV-1 antibody using particle agglutination (PA) or chemiluminescent enzyme immunoassay (EIA) with western blotting (WB) and/or polymerase chain reaction (PCR) confirmation in all pregnant women [14, 15]. The screening test is performed during early-to-middle pregnancy (up to around 30 weeks of pregnancy). If the screening test is negative, the pregnant woman is judged to be a non-infected person. If the screening test is positive, the individual might be a carrier; therefore, a confirmation test via WB method is always performed. It is recommended that the PCR method be performed if the WB method is suspended, and it was listed as part of the national health insurance in April 2016. The PCR-positive rate of the decision holder was estimated to be about 20%.

Serological screening via EIA or PA tests has been used to detect HTLV-1 antibodies in all pregnant women in Japan at the expense of the Japanese public fund since September 2010. If the result of the screening is positive, confirmatory testing by WB can be performed to eliminate false-positive reactions, which is covered by the Universal Health Insurance system. This is important because a considerable number of tests had a false-positive result by EIA or PA screening tests. Diagnosis as an HTLV-1 carrier can be determined only after a confirmatory test (WB test); however, a polymerase chain reaction (PCR) test is also available (at one’s own expense) to further refine the diagnosis. In uncertain serological consultations, PCR analysis can provide a definitive diagnosis of infection.

Diagnosis as an HTLV-1 carrier can usually be determined after the confirmation test (WB test) following a serological screening test performed for all women during pregnancy in Japan since September 2010. According to the Guidelines for Obstetrical Practice in Japan published in 2011, these methods are advised for HTLV-1-positive pregnant women to prevent vertical transmission [14, 15].

### Study design and data collection

**Fig. 1:**
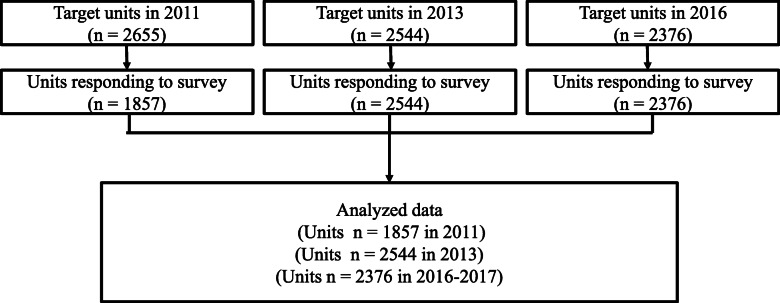
Study data flow

**Fig. 2:**
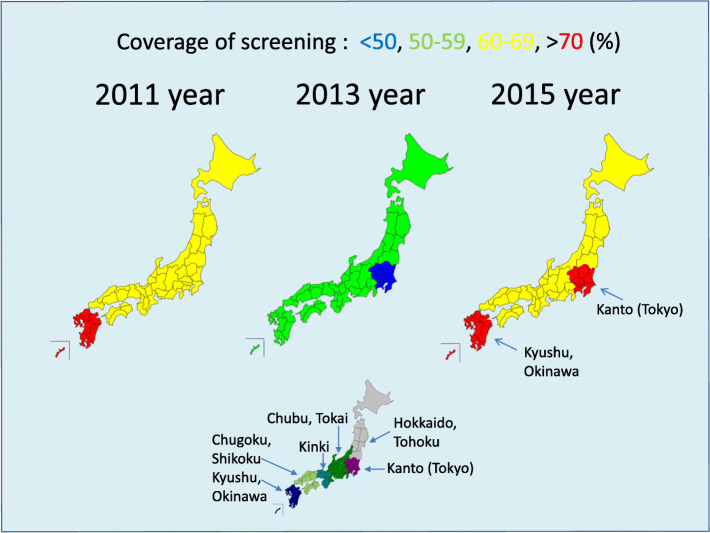
Map of screening proportion of pregnant women by region in Japan (%). The map was created by software (Shiro-chizu nuri-nuri) using our survey data and public vital statistics in Japan

This was a retrospective repeated cross-sectional study. We used data from three surveys for HTLV-1-antibody-positive pregnant women performed by the Japan Association of Obstetricians and Gynecologists (JAOG) [16–18]. The three questionnaire surveys were administered in 2011, 2013, and 2016 (April 2016–March 2017) by the Japanese Association of Gynecologists including head obstetrics and gynecologists in all 47 prefectures in Japan (Fig. 2). Target medical facilities that performed the screening test certified by the Japanese Association of Gynecologists were included in the survey. Data from hospitals included information of the region, not the prefecture, to protect the hospital information. Japan had six regions as follows; Hokkaido and Tohoku, Kanto (including Tokyo), Chubu and Tokai, Kinki, Chugoku and Shikoku, and Kyusyu. Three questionnaire surveys were conducted by the JAOG in 2011, 2013, and 2016 (April 2016 to March 2017). All medical facilities that handled the delivery of the questionnaire surveys were included in the survey. Analyses from each survey were reported elsewhere [16–18]. Outcomes for evaluation included the coverage of screening (number of persons who conducted the screening test/number of total pregnant cases), the number of positive persons identified by these tests, and the proportion of positive persons to the number of persons who performed the tests. Unfortunately, the survey contained no data regarding the total number of pregnant women in a hospital. Alternatively, we calculated the total number of pregnant women by region from vital statistics (supplement file 1) [19]. Then, we calculated the coverage of screening by region but not by hospital. We originally drew the map in Japan with our survey data and vital statistics in public by free license software. (Shiro-chizu nuri-nuri: https://n.freemap.jp/) (figure1) The study was approved by the ethics committee of the JAOG. We have reported this report in accordance with STROBE statement (supplement file 2) [20].

### Statistical analysis

The number of target facilities, number of screening facilities, number of people who carried out the test method, and number of positive individuals are summarized according to annual regional area and type of units. We performed multivariate Poisson’s regression with a generalized estimating equation to examine the impact on these covariates [21]. Rate ratios, 95% confidence intervals, and *p*-values were calculated. Denominator of the outcome variable as pregnant women or number of people who carried out the test method were included as the offset in the model. Because this research was exploratory, the priorities of outcomes were not set in the analysis. The significance level of *p*-values was set to 5% on both sides as supplementary information. All data were analyzed using SAS version 9.4.

## Results

Numbers of target facilities changed yearly: 1857 in 2011, 2544, in 2013, and 2376 in 2016 (Fig. 1). However, the configuration of facilities was similar in the 2013 and 2016 surveys. Moreover, the regional composition was similar in all surveys. The numbers of facilities that implemented screening changed yearly: 1779 facilities (95.8%) in 2011, 1367 (53.7) in 2013, and 1742 (73.3) in 2016 (Table 1). The change in screening coverage is shown by regions on a map of Japan (Fig. 2). The mean number of screening test participants increased per facility, but the median increased or decreased. The number of positive individuals was similar. For the WB method, the number of practitioners and number of positive individuals tended to decrease after 2011, with approximately one positive person per center. The PCR method had a high mean number of practitioners in 2016, but because only one facility performed PCR with many practitioners, the median did not change. The number of positive individuals identified by PCR was approximately 0.5 at this facility (Table 2).

**Table 1:** Characteristics of hospitals and clinics, n (%)

Year	2011			2013			2016		
	Target	Screening	S/T (%)^a^	Target	Screening	S/T (%)^a^	Target	Screening	S/T (%)^a^
Total of units	1857	1779	95.8	2544	1367	53.7	2376	1742	73.3
Units									
High-volume hospital	258 (13.9)	252	97.7	1091 (42.9)	572	52.4	1041 (43.8)	731	70.2
Middle- and low-volume hospitals and clinics	1599 (86.1)	1527	95.5	1453 (57.1)	795	54.7	1335 (56.2)	1011	74.6
Region									
Hokkaido, Tohoku	225 (12.1)	217	96.4	305 (12.0)	156	51.1	275 (11.6)	190	69.1
Kanto	459 (24.7)	443	96.5	661 (26.0)	314	50.5	634 (26.7)	441	69.6
Chubu, Tokai	367 (19.8)	352	95.9	494 (19.4)	273	55.3	456 (19.2)	340	74.6
Kinki	284 (15.3)	274	96.5	412 (16.2)	236	57.3	385 (16.2)	286	74.3
Chugoku, Shikoku	209 (11.3)	198	94.7	270 (10.6)	154	57.0	247 (10.4)	182	73.7
Kyusyu, Okinawa	313 (16.9)	295	94.2	402 (15.8)	234	58.2	379 (16.0)	296	78.1

^a^S/T (%): (n of screening hospitals and clinics/n of target hospitals and clinics) × 100

**Table 2:** Annual change in the number of positive individuals by test per unit

Target	n of units	2011	2013	2016
		1857	2544	2375
Screening	n of units (%)	1779 (95.8)	1367 (53.7)	1742 (73.3)
Tested	Mean (SD) Median (interquartile)	388.9 (387.0) 309.0 (175–502)	397.8 (311.6) 286 (194–502)	400.2 (348.2) 320.5 (195–509)
Positive	Mean (SD) Median (interquartile)	2.0 (20.4) 0 (0–1)	1.7 (14.7) 0 (0–2)	1.6 (12.2) 0 (0–2)
WB	n of units (%)	803 (43.2)	860 (33.8)	1699 (71.5)
Tested	Mean (SD) Median (interquartile)	13.1 (73.1) 1 (1–3)	5.9 (47.2) 1 (0–3)	5.9 (44.2) 0 (0–2)
Positive	Mean (SD) Median (interquartile)	1.2 (3.9) 1 (0–1)	1.0 (1.7) 1 (0–1)	0.5 (1.4) 1 (0–1)
PCR	n of units (%)	816 (43.9)	78 (3.1)	255 (10.7)
Tested	Mean (SD) Median (interquartile)	2.5 (28.5) 0 (0–0)	2.4 (2.5) 1 (1–3)	20.5 (112.0) 0 (0–1)
Positive	Mean (SD) Median (interquartile)	0.2 (3.6) 0 (0–0)	0.5 (0.9) 0 (0–1)	0.6 (6.3) 0 (0–0)

By multivariate analysis, areas (Kanto and Kinki) and facility types showed slightly increased screening coverage although screening coverage in other areas (Kanto and Chubu) decreased. Positive rates were decreased in some areas (Hokkaido/Tohoku, Kanto, and Chugoku/Shikoku) and positive identification increased by facility type. Numbers of WB performed were decreased in 2016 and the positive identification rate was lower in 2016 for all areas; however, facility types were increased. The number of PCR participants was markedly increased in 2016 in Kanto and Kinki; however, the facility types were decreased. The positive identification rate for PCR decreased in all areas (except the Chubu region) but facility types were increased (Tables 3, 4 and 5).

**Table 3:** Screening, rate ratio (95% CI), *p*-value

	Screening				Screening positive			
	Rate ratio	95%CI		*P*-value	Rate ratio	95%CI		*P*-value
Year								
2011	Reference				Reference			
2013	0.96	0.91	1.02	0.1781	0.74	0.42	1.30	0.2876
2016	1.00	0.95	1.05	0.9242	0.69	0.38	1.27	0.2329
Regions								
Hokkaido, Tohoku	1.20	1.04	1.37	0.0119	0.21	0.13	0.33	<.0001
Kanto	0.55	0.48	0.62	<.0001	0.30	0.17	0.52	<.0001
Chubu, Tokai	0.77	0.68	0.87	<.0001	0.43	0.17	1.06	0.0670
Kinki	0.89	0.79	1.02	0.0928	0.54	0.25	1.16	0.1142
Chugoku, Shikoku	1.33	1.15	1.53	0.0001	0.22	0.14	0.35	<.0001
Kyusyu, Okinawa	Reference				Reference			
Units								
High-volume hospital	1.18	1.11	1.26	<.0001	1.56	1.02	2.38	0.0386
Middle- and low-volume hospitals and clinics	Reference

**Table 4:** Western blot, rate ratio (95% CI), *p*-value

	WB				WB positive			
	Rate ratio	95%CI		*P*-value	Rate ratio	95%CI		*P*-value
Years								
2011	Reference				Reference			
2013	0.57	0.27	1.21	0.1435	0.87	0.66	1.14	0.3168
2016	0.54	0.33	0.88	0.0145	0.59	0.45	0.77	<.0001
Regions								
Hokkaido, Tohoku	1.00	0.46	2.20	0.9982	0.30	0.22	0.40	<.0001
Kanto	0.66	0.34	1.27	0.2140	0.29	0.22	0.39	<.0001
Chubu, Tokai	0.88	0.36	2.12	0.7744	0.25	0.19	0.33	<.0001
Kinki	0.85	0.46	1.59	0.6198	0.35	0.27	0.46	<.0001
Chugoku, Shikoku	0.67	0.29	1.52	0.3385	0.27	0.21	0.36	<.0001
Kyusyu, Okinawa	Reference				Reference			
Units								
High-volume hospital	0.63	0.36	1.08	0.0907	1.59	1.19	2.13	0.0019
Middle- and low-volume hospital and clinic	Reference

**Table 5:** PCR, rate ratio (95% CI), *p*-value

	PCR				PCR positive			
	Rate ratio	95%CI		*P*-value	Rate ratio	95%CI		*P*-value
Years								
2011	Reference				Reference			
2013	2.44	0.81	7.32	0.1121	0.96	0.17	5.50	0.9645
2016	16.06	6.02	42.84	<.0001	1.54	0.29	8.23	0.6137
Regions								
Hokkaido, Tohoku	5.36	0.71	40.40	0.1032	0.13	0.03	0.55	0.0051
Kanto	1.40	0.41	4.79	0.5908	0.09	0.03	0.35	0.0004
Chubu, Tokai	1.83	0.34	9.77	0.4819	0.93	0.14	6.16	0.9410
Kinki	3.68	1.10	12.26	0.0340	0.12	0.04	0.43	0.0011
Chugoku, Shikoku	3.42	0.70	16.78	0.1297	0.10	0.02	0.44	0.0022
Kyusyu, Okinawa	Reference				Reference			
Units								
High-volume hospitals	0.14	0.04	0.54	0.0045	10.93	2.94	40.59	0.0004
Middle- and low-volume hospitals and clinics	Reference							

## Discussion

The study evaluated the national implementation of HTLV-1 screening in Japan. To the best of our knowledge, this is the first nationwide routine screening of pregnant women for HTLV-1 infection. The HTLV-1 screening program in Japan was almost fully implemented but variations in screening tests were observed. Endemic or non-endemic countries or areas might have different perspectives regarding the need to introduce a nationwide screening program, but in countries or areas where HTLV-1 is endemic, antenatal screening is likely to contribute to a reduction in associated diseases. Most previous reports of nationwide screening estimated the incidence or prevalence for research purposes but not the implementation of screening as a routine health service program [22, 23].

The National Screening Committee in the UK previously discussed a national HTLV-1 screening program but the committee did not recommend implementing the screening because the UK had a low prevalence of HTLV-1 infection and there was a low risk for infected infants to develop a serious illness. The Committee reiterated its previous conclusions in 2017 [24]. Recently, a cost-effectiveness study of HTLV-1 screening in the UK was reported [25]. The analysis used a highly conservative model of transmission and disease attribution. They reported that a screening program to identify HTLV-1 carriers to reduce transmission was potentially cost-effective in the UK. Therefore, our study findings might provide information useful for implementing a national screening program in other countries.

The implementation of HTLV-1 screening was conducted nationwide after its introduction in 2011. There was no consistent overall trend because differences were observed by region and facility type for each examination. The number of people per facility varied widely. There were about 200–500 people per facility per quartile in the analysis. This may have been influenced by the local infection rate [11, 12] and local government’s commitment to HTLV-1. Kyushu area was the most endemic area in Japan [15, 16]. Facility types were associated with the implementation of the screening and testing. Local governments might the change role of facilities for screening and testing to optimize medical resources. 2013 year was a time of transition on the implementation. It is hoped that the dissemination, inspection, and follow-up of this study will consider regional and facility characteristics. To the best of our knowledge, this is the first evaluation of a nationwide HTLV-1 screening program. Our findings are relevant to the implementation of similar screening programs.

### Limitations

This study performed an integrated analysis of three questionnaires and had some limitations. First, the survey was institutional and there was no information about individuals (pregnant women or children). The data only provided counts of categories by individuals. Data of pregnant women were obtained from vital statistics and only by region. Second, some unmeasured confounders and bias effects of individual factors could not be adjusted for. Third, because the questionnaire was a survey of the facility, there was the potential for response bias. Fourth, the time intervals for each survey were different. Finally, HTLV-1 infection rates vary by region, which may be reflected in the results. Although information on the infection rate in each region is limited, previous studies reported that the infection rate of HTLV-1 was high in Kyushu and Okinawa. Multivariate analysis assumes that the factors evaluated have been adjusted from the observed information; thus, changes are only reported for the reference group.

## Conclusion

The nationwide screening program for HTLV-1 in Japan was almost fully implemented. However, variations in screening tests were observed during its implementation. Thus, some incentives are needed to encourage proper implementation across all regions.

## Supplementary information

**Additional file 1.** Screening and pregnant women by region in Japan, n (%).Number of screening and Birth and still birth in population by region (Hokkaido and Tohoku, Kanto, Chubu and Tokai, Kinki, Chugoku and Shikoku, Kyusyu and Okinawa) and by year (2011, 2013, 2015).

**Additional file 2.** STROBE statement. Checklist table of STROBE statement for the manuscript.

## Data Availability

The data in this study are not publicly available due to data security agreements with JAOG, but data are available from the corresponding author upon reasonable request with permission from JAOG.

## Abbreviations

EIA: Enzyme immunoassay
HTLV-1: Human T-lymphotropic virus type 1
JAOG: Japan Association of Obstetricians and Gynecologists
MHLW: Ministry of Health, Labor and Welfare
MTCT: Mother-to-child transmission
WB: Western blotting
PA: Particle agglutination
PCR: Polymerase chain reaction
STROBE: Strengthening the reporting of observational studies in Epidemiology
UK: United Kingdom

## References

[1] Fuchi N, Miura K, Tsukiyama T, Sasaki D, Ishihara K, Tsuruda K, Hasegawa H, Miura S, Yanagihara K, Masuzaki H (2018). Natural course of human T-cell leukemia virus type 1 Proviral DNA levels in carriers during pregnancy. J Infect Dis.

[2] LaGrenade L, Hanchard B, Fletcher V, Cranston B, Blattner W (1990). Infective dermatitis of Jamaican children: a marker for HTLV-I infection. Lancet..

[3] Martin F, Tagaya Y, Gallo R (2018). Time to eradicate HTLV-1: an open letter to WHO. Lancet..

[4] Willems L, Hasegawa H, Accolla R, Bangham C, Bazarbachi A, Bertazzoni U, Carneiro-Proietti AB, Cheng H, Chieco-Bianchi L, Ciminale V, Coelho-Dos-Reis J, Esparza J, Gallo RC, Gessain A, Gotuzzo E, Hall W, Harford J, Hermine O, Jacobson S, Macchi B, Macpherson C, Mahieux R, Matsuoka M, Murphy E, Peloponese JM, Simon V, Tagaya Y, Taylor GP, Watanabe T, Yamano Y (2017). Reducing the global burden of HTLV-1 infection: an agenda for research and action. Antivir Res.

[5] Boostani R, Sadeghi R, Sabouri A, Ghabeli-Juibary A (2018). Human T-lymphotropic virus type I and breastfeeding; systematic review and meta-analysis of the literature. Iran J Neurol.

[6] Percher F, Jeannin P, Martin-Latil S, Gessain A, Afonso PV, Vidy-Roche A, Ceccaldi PE (2016). Mother-to-Child Transmission of HTLV-1 Epidemiological Aspects, Mechanisms and Determinants of Mother-to-Child Transmission. Viruses.

[7] Moriuchi H, Masuzaki H, Doi H, Katamine S (2013). Mother to-child transmission of human T-cell lymphotropic virus type1. Pediatr Infect Dis J.

[8] Rosadas C, Taylor GP (2019). Mother-to-Child HTLV-1 Transmission: Unmet Research Needs. Front Microbiol.

[9] Tsuji Y, Doi H, Yamabe T, Ishimaru T, Miyamoto T, Hino S (1990). Prevention of mother-to-child transmission of human T-lymphotropic virus type-I. Pediatrics.

[10] Eshima N, Iwata O, Iwata S, Tabata M, Higuchi Y, Matsuishi T, Karukaya S (2009). Age and gender specific prevalence of HTLV-1. J Clin Virol.

[11] Yamada T, Togashi T, Tsutsumi H, Imamura M, Okubo H, Okabe M, Takamuro N, Tashiro K, Yano K, Yamamoto N, Hirakawa Y, Minakami H (2014). Prevalence of human T-lymphotropic virus type 1 carriers among pregnant women in Hokkaido, Japan. Microbiol Immunol.

[12] Satake M, Iwanaga M, Sagara Y, Watanabe T, Okuma K, Hamaguchi I (2016). Incidence of human T-lymphotropic virus 1 infection in adolescent and adult blood donors in Japan: a nationwide retrospective cohort analysis. Lancet Infect Dis.

[13] Satake M, Yamaguchi K, Tadokoro K (2012). Current prevalence of HTLV-1 in Japan as determined by screening of blood donors. J Med Virol.

[14] Minakami H, Hiramatsu Y, Koresawa M, Fujii T, Hamada H, Iitsuka Y, Ikeda T, Ishikawa H, Ishimoto H, Itoh H, Kanayama N, Kasuga Y, Kawabata M, Konishi I, Matsubara S, Matsuda H, Murakoshi T, Ohkuchi A, Okai T, Saito S, Sakai M, Satoh S, Sekizawa A, Suzuki M, Takahashi T, Tokunaga A, Tsukahara Y, Yoshikawa H. Japan Society of Obstetrics and Gynecology, Japan Association of Obstetricians and Gynecologists, (2011) Guidelines for obstetrical practice in Japan: Japan Society of Obstetrics and Gynecology (JSOG) and Japan Association of Obstetricians and Gynecologists (JAOG) 2011 edition. J Obstet Gynaecol Res. 37:1174–97. 10.1111/j.1447-0756.2011.01653.x 21917078

[15] Itabashi K, Miyazawa T, Sekizawa A, Tokita A, Saito S, Moriuchi H, Nerome Y, Uchimaru K, Watanabe T (2020). A Nationwide antenatal human T-cell leukemia virus Type-1 antibody Screening in Japan. Front Microbiol.

[16] Suzuki S, Tanaka M, Matsuda H, Tsukahara Y, Kuribayashi Y, Nakai A, Miyazaki T, Kamiya N, Sekizawa A, Mizutani N, Kinoshita K (2015). Prevalence of human T-cell leukemia virus type 1 carrier in Japanese pregnant women in 2013. J Clin Med Res.

[17] Suzuki S, Tanaka M, Matsuda H, Tsukahara Y, Kuribayashi Y, Gomibuchi H, Miyazaki R, Kamiya N, Nakai A (2014). Kinoshita K; Japan Association of Obstetricians and Gynecologists. Current status of HTLV-1 carrier in Japanese pregnant women. J Matern Fetal Neonatal Med.

[18] Suzuki S, Tanaka M, Matsuda H, Tsukahara Y, Kuribayashi Y, Gomibuchi H, Miyazaki R, Kamiya N, Nakai A, Kinoshita K (2014). Instruction of feeding methods to Japanese pregnant women who cannot be confirmed as HTLV-1 carrier by western blot test. J Matern Fetal Neonatal Med.

[19] von Elm E, Altman DG, Egger M, Pocock SJ, Gøtzsche PC (2007). Vandenbroucke JP; STROBE initiative. Strengthening the reporting of observational studies in epidemiology (STROBE)statement: guidelines for reporting observational studies. BMJ..

[20] Ministry of Health, Labour and Welfare, Vital statistics in Japan https://www.mhlw.go.jp/english/database/db-hw/vs01.html.

[21] Shoukri MM, Chaudhary MA. Analysis of correlated data with SAS and R. 3rd ed: Chapman & Hall/CRC, Taylor & Francis group; 2007.

[22] Ribeiro MA, Martins ML, Teixeira C, Ladeira R, Oliveira Mde F, Januário JN, Proietti FA, Carneiro-Proietti AB (2012). Blocking vertical transmission of human T cell lymphotropic virus type 1 and 2 through breastfeeding interruption. Pediatr Infect Dis J.

[23] Rosadas C, Malik B, Taylor GP, Puccioni-Sohler M (2018). Estimation of HTLV-1 vertical transmission cases in Brazil per annum. PLoS Negl Trop Dis.

[24] UK National Screening Committee. Antenatal Screening for HTLV infection. Available online at: https://legacyscreening.phe.org.uk/policydb_download.php?doc=704 (accessed June 13, 2020).

[25] Malik B, Taylor GP (2019). Can we reduce the incidence of adult T-cell leukaemia/ lymphoma? Cost-effectiveness of human T-lymphotropic virus type 1 (HTLV-1) antenatal screening in the United Kingdom. Br J Haematol.

