# Correction to: Implementation of nationwide screening of pregnant women for HTLV-1 infection in Japan: analysis of a repeated cross-sectional study

**DOI:** 10.1186/s12889-020-09314-z

**Published:** 2020-08-03

**Authors:** Naohiro Yonemoto, Shunji Suzuki, Akihiko Sekizawa, Shinichi Hoshi, Yoko Sagara, Kazuo Itabashi

**Affiliations:** 1 Department of Neuropsychopharmacology, National Center of Neurology and Psychiatry, 4-1-1, Ogawahigashi, Kodaira, Tokyo, Japan; 2 Department of Public Health, Juntendo University School of Medicine, 2-1-1 Hongo, Bunkyo, Tokyo, Japan; 3 Japan Association of Obstetricians and Gynecologists, 14 Yahatacho, Ichigaya-Hachimanmachi, Shinjuku-ku, Tokyo, 162-0844 Japan; 4 Department of Obstetrics and Gynecology, Japanese Red Cross Katsushika Maternity Hospital, 5-11-12 Tateishi, Katsushika-ku, Tokyo, 124-0012 Japan; 5 Department of Obstetrics and Gynecology, Showa University School of Medicine, 1-5-8 Hatanodai, Shinagawa-ku, Tokyo, 142-8666 Japan; 6 Department of Pediatrics, Showa University School of Medicine, 1-5-8 Hatanodai, Shinagawa-ku, Tokyo, 142-8666 Japan

**Correction to: BMC Public Health 20, 1150 (2020)**

**https://doi.org/10.1186/s12889-020-09258-4**

Following publication of the original article [1], the authors identified an error in the author name of Kazuo Itabashi

- The incorrect author name is: Kazuo Itahashi
- The correct author name is: Kazuo Itabashi

The author group has been updated above and the original article [1] has been corrected.
