# Correction to: Implementation of nationwide screening of pregnant women for HTLV-1 infection in Japan: analysis of a repeated cross-sectional study

**DOI:** 10.1186/s12889-021-11049-4

**Published:** 2021-05-21

**Authors:** Naohiro Yonemoto, Shunji Suzuki, Akihiko Sekizawa, Shinichi Hoshi, Yoko Sagara, Kazuo Itabashi

**Affiliations:** 1 Department of Neuropsychopharmacology, National Center of Neurology and Psychiatry, 4-1-1, Ogawahigashi, Kodaira, Tokyo, Japan; 2 Department of Public Health, Juntendo University School of Medicine, 2-1-1 Hongo, Bunkyo, Tokyo, Japan; 3 Japan Association of Obstetricians and Gynecologists, 14 Yahatacho, Ichigaya-Hachimanmachi, Shinjuku-ku, Tokyo, 162-0844 Japan; 4 Department of Obstetrics and Gynecology, Japanese Red Cross Katsushika Maternity Hospital, 5-11-12 Tateishi, Katsushika-ku, Tokyo, 124-0012 Japan; 5 Department of Obstetrics and Gynecology, Showa University School of Medicine, 1-5-8 Hatanodai, Shinagawa-ku, Tokyo, 142-8666 Japan; 6 Department of Pediatrics, Showa University School of Medicine, 1-5-8 Hatanodai, Shinagawa-ku, Tokyo, 142-8666 Japan

**Correction to: BMC Public Health 20, 1150 (2020)**

**https://doi.org/10.1186/s12889-020-09258-4**

The authors of this study [1] would like to amend it and clarify that description of the results in abstract and body of the paper and references were incorrect. This amendment does not cause any changes to the substantive contents of the article since errors in the description of the results in the table, no change in the table.

1). Reference 19 and 20 is reversed. The following description is correct.

19. Ministry of Health, Labour and Welfare, Vital statistics in Japan https://www.mhlw.go.jp/english/database/db-hw/vs01.html

20. von Elm E, Altman DG, Egger M, Pocock SJ, Gtzsche PC. Vandenbroucke JP; STROBE initiative. Strengthening the reporting of observational studies in epidemiology (STROBE)statement: guidelines for reporting observational studies. BMJ. 2007;335:8068.

2). In abstract, the descriptions do not match the results in the table 3 and 5. The following description is correct.

Results: Numbers of target facilities changed yearly: 1857 in 2011, 2544 in 2013, and 2376 in 2016. The mean number of screening-test participants increased per facility, but the median increased or decreased. The mean number of positive individuals identified decreased. Multivariate analysis results revealed the number of screenings was reduced in Kanto and Chubu/Tokaiareas, although some areas (Hokkaido/Tohoku and Chugoku/Shikoku) and high volume in facility types increased. Regarding the positive rates, some areas (Hokkaido/Tohoku, Kanto, and Chugoku/Shikoku) exhibited decreases or increases by facility type. The number of western blotting (WB) implementations decreased in 2016, positive rates identified by WB decreased in 2016 andin all areas, and the number of facility types increased. The number of PCR participants increased in 2016 andin Kinki, but a decrease in facility type was observed. Positive rates were decreased in all areas (except Chubu/Tokaiarea) but facility types were increased.

3) (P3) Results: the descriptions do not match the results in the table 3 and 5. The following description is correct.

By multivariate analysis, some areas (Hokkaido/Tohoku and Chugoku/Shikoku) and facility types showed increased screening coverage although screening coverage in other areas (Kanto and Chubu) decreased. Positive rates were decreased in some areas (Hokkaido/Tohoku, Kanto, and Chugoku/Shikoku) and positive identification increased by facility type. Numbers of WB performed were decreased in 2016 and the positive identification rate was lower in 2016 and inall areas; however, facility types were increased. The number of PCR participants was markedly increased in 2016 and inKinki;
