# Supplementary material for: Implementation of nationwide screening of pregnant women for HTLV-1 infection in Japan: analysis of a repeated cross-sectional study
Source: BMC Public Health. 2020 Jul 22;20:1150. doi: 10.1186/s12889-020-09258-4 (PMC7374850; doi:10.1186/s12889-020-09258-4)
Supplement: Supplementary file 1 — Additional file 1. Screening and pregnant women by region in Japan, n (%).Number of screening and Birth and still birth in population by region (Hokkaido and Tohoku, Kanto, Chubu and Tokai, Kinki, Chugoku and Shikoku, Kyusyu and Okinawa) and by year (2011, 2013, 2015). [file 12889_2020_9258_MOESM1_ESM.docx]

| Region | 2011, number | | | 2013, number | | | 2015, number | | |
| --- | --- | --- | --- | --- | --- | --- | --- | --- | --- |
|  | Screening | Birth and  still birth in population | % | Screening | Birth and  still birth in population | % | Screening | Birth and  still birth in population | % |
| Hokkaido, Tohoku | 67906 | 109596 | 62.0 | 54917 | 107153 | 51.3 | 64660 | 98670 | 65.5 |
| Kanto | 215103 | 353263 | 60.9 | 162633 | 350616 | 46.4 | 225992 | 338625 | 66.7 |
| Chubu,  Tokai | 130166 | 201484 | 64.6 | 107877 | 194921 | 55.3 | 133483 | 182447 | 73.2 |
| Kinki | 107634 | 177190 | 60.7 | 93805 | 171913 | 54.6 | 113018 | 162976 | 69.3 |
| Chugoku, Shikoku | 63535 | 96964 | 65.5 | 47696 | 93712 | 50.9 | 60874 | 87183 | 69.8 |
| Kyusyu, Okinawa | 107564 | 137927 | 78.0 | 76824 | 135538 | 56.7 | 99099 | 127935 | 77.5 |
| Total | 691908 | 1076424 | 64.3 | 543752 | 1053853 | 51.6 | 697126 | 997836 | 69.9 |
